# Solidification of hydatid cyst fluid with an injectable chitosan/carboxymethylcellulose/β-glycerophosphate hydrogel for effective control of spillage during aspiration of hydatid cysts

**DOI:** 10.1007/s40204-018-0082-5

**Published:** 2018-02-19

**Authors:** Mostafa D. A. Azadi, Shadi Hassanjili, Khalil Zarrabi, Bahador Sarkari

**Affiliations:** 1 Department of Chemical Engineering, School of Chemical and Petroleum Engineering, Shiraz University, Shiraz, Iran; 2 Department of Cardiovascular Surgery, School of Medicine, Shiraz University of Medical Sciences, Shiraz, Iran; 3 Department of Parasitology and Mycology, School of Medicine, Shiraz University of Medical Sciences, Shiraz, Iran

**Keywords:** Hydatid cyst, Injectable hydrogels, Thermosensitive polymers, Chitosan, Carboxymethyl cellulose, Response surface methodology, Central composite design

## Abstract

Cystic echinococcosis (CE)/hydatid cyst is one of the most important helminthic diseases in the world. The treatment of hydatid cyst ranges from surgical intervention to chemotherapy, although the efficacy of chemotherapy is still unclear. Postoperative complication which results from the spillage of cysts during surgical operation is one of the most important concerns in surgical treatment of hydatid cyst. The aim of the current study was to solidify the hydatid cyst fluid (HCF) with an injectable and thermosensitive chitosan (CS)/carboxymethyl cellulose (CMC)/β-glycerol phosphate (BGP) hydrogel for effective control of spillage during the aspiration of hydatid cysts. Fourier-transform infrared spectroscopy (FTIR), scanning electron microscopy (SEM), water uptake, rheological analysis, and Alamar Blue cytotoxicity assay were employed to characterize the hydrogel. A five level with three times replication at the central point using a central composite design (CCD), which is a response surface methodology (RSM), was used to optimize the experimental conditions. Assessment of the produced hydrogel showed that the intermolecular interactions of amino groups of chitosan and hydrogen groups of CMC were correctively established and appreciable swelling with a good strength was obtained. Hydrogels morphology had a porous structure. Rheological analysis showed that CS/CMC/BGP blends had a phase transition (32–35 °C) of sol–gel close to the body temperature. Alamar Blue cytotoxicity assay showed that CS (1.75%)/CMC (1.4%)/BGP (2.9%) had IC50 values of 0.598, 0.235 and 0.138 (µg/µL) for 24, 48 and 72 h, which indicated that the produced polymer solution had no significant cytotoxic effect for human fibroblast cell line. In vitro injection of the polymer solution of CS/CMC/BGP with CS/CMC ratio of 1.75/1.4 was done on HCF (1 mL polymer solution to 3 mL of HCF) at 37 °C with a final concentration of 2.9% for BGP resulting in solidification of HCF in less than 45 min.

## Introduction

Cystic echinococcosis/hydatid cyst is one of the most important and oldest known human zoonotic diseases in the world. The disease is caused by the larval stage of *Echinococcus granulosus*. Hydatid cyst disease is a major endemic health problem in certain areas of the world such as Asia, Europe, South America, the Middle East, Australia and New Zealand (Dervenis et al. 2005; Rahimi et al. 2011; Smego et al. 2003). The disease has been reported worldwide, but the highest proportion of contamination is in the Mediterranean region, including Iran (Moghadar et al. 1992). Dogs and other canines are considered to be definitive hosts. Human, sheep, cattle, camel, etc. are considered to be the main intermediate hosts. Humans become infected by ingestion of vegetable, food and water contaminated by excreted parasite eggs through dog feces. Embryo eggs (oncosphere) hatch and go through the bloodstream to various tissues (liver, lung, spleen, brain, etc.) and gradually form the hydatid cyst (El Malki et al. 2010; Gillespie et al. 2003). Hydatid cyst fluid is clear with a specific gravity of 1.007–1.015 g/cm^3^, containing albumin and other proteins, 0.5% salt, phosphate and calcium sulfate salts, sugar, fat and other substances. This liquid has a pH of 7.2–7.4. Hydatid cyst fluid is free of bacteria, but is a nurturing environment for bacterial growth (Rigano et al. 2001). The current treatments for hydatid cyst include chemotherapy, surgery and percutaneous drainage, known as PAIR (puncture, aspiration, injection of scolicidal agents and respiration). A combination of these treatments can be a fairly effective method in its treatment (Smego et al. 2003; Smego and Sebanego 2005; Yagci et al. 2005). If human hydatid cyst ruptures due to injury or any other reasons, parasite materials (protoscoleces) spread in the adjacent viscera and any protoscolex can make a potential daughter cyst and possibly anaphylaxis shock or occasionally death (Hosseini et al. 2006; Mansourian et al. 2009). Due to the existing problems in the treatments of hydatid cyst, solidification of hydatid cyst fluid can eliminate the risk of daughter cyst formation. For solidification, injectable and thermosensitive hydrogels as gelling agents can be a suitable choice. Hydrogels are three-dimensional networks consisting of high molecular weight hydrophilic natural or synthetic polymers, water and cross-linking agents. They swell considerably in aqueous environments and have an extraordinary capacity to absorb water or physiological aqueous solutions into their network structure, though they are not soluble in neither of them. Hydrophilic groups in the structure of the hydrogels help them to absorb water and swell. These can be anionic, cationic or neutral (Bhattarai et al. 2010; Zhang et al. 2013). Swelling behavior, structure and morphology are important characteristics of the hydrogels that heavily depend on the chemical nature of monomers. The amount of absorbed fluid in the hydrogels depends on the formulation, impurities and salt content. Because of the remarkable characteristics, including physical and chemical properties, high biocompatibility, versatility in fabrication, low surface tension and similarity to biological tissues, hydrogels have emerged as promising materials (Annabi et al. 2010; Peppas et al. 2006; Slaughter et al. 2009). and have been used in a number of biomedical applications, such as wound dressing (Miguel et al. 2014), tissue engineering (Thi-Hiep et al. 2013) and drug delivery (Ruel-Gariépy et al. 2004). Hydrophilic properties and swelling of the hydrogels are due to functional groups, such as –OH, –COOH, –CONH_2_ and –SO_3_H in their polymer chains and their insolubility is because of its cross-linked network (Ganji et al. 2010; Hamidi et al. 2008). Gel formation can occur physically or chemically. A physical gel formation is due to stimuli-responsive sensitive groups (thermo, pH, pressure, electrical field, etc.) or polymer chain association and chemically cross-linked gel formation can be acquired by radical polymerization with appropriate initiators and cross-linking agents, especially non-toxic high energy irradiation (gamma and electron beam and UV light) for some biomedical applications (Hennink and Van Nostrum 2012). Among physically cross-linked hydrogels, chitosan (CS)-based systems are particularly interesting due to their ability to form solutions with neutral pH at low temperature, which gel at body temperature. This can be achieved by adding a weak base such as β-glycerophosphate (BGP) to an acidic solution of CS (Ruel-Gariepy et al. 2000; Tahrir et al. 2015). Chitosan (β-(1 → 4)-linked 2-acetamido-2-deoxy d-glucopyranose and 2-amino-2-deoxy-d-glucopyranose) is a natural polymer obtained from partial deacetylation of chitin. It is derived from cell walls of fungi, the exoskeletons of insects, crustaceans, such as shrimp and crabs (Casettari et al. 2012; Rinaudo 2006). The unique properties of chitosan such as non-toxicity (Tang et al. 2014), antimicrobial properties (Jarry et al. 2001), biocompatibility, biodegradability and gelation properties (Ao et al. 2011) make it useful in various fields such as biomedical applications (Rokhade et al. 2007), water filtration (Cooper et al. 2013) and so on. While CS/BGP gels have been used in several applications (Ruel-Gariepy et al. 2000; Chenite et al. 2000; Tahrir et al. 2015), the slowness of gelation and the weakness of mechanical properties after gelation limit their performance. Ceccaldi et al. (2017) prepared strong injectable chitosan-thermosensitive hydrogels for cell therapy and tissue engineering purposes, by combining sodium hydrogen carbonate with β-glycerophosphate and studied the influence of gelling agent concentration on the mechanical and physical properties of hydrogels. Dai et al. (2018) achieved an injectable, biodegradable and thermosensitive hydrogel based on CS/BGP loaded with poly(lactic-*co*-glycolic acid) (PLGA) nanoparticles (NPs-CS/BGP) which showed a rapid transition from solution to gel as temperature increased, a porous structure and a long-term in vitro release profile. As polymer blending is an important method for improving the physical properties of polymeric materials, the main purpose of this study is to prepare injectable and thermosensitive physically cross-linked chitosan (CS)/carboxymethylcellulose (CMC)/β-glycerol phosphate (BGP) blend hydrogels. CMC is a natural anionic polymer and is one of the cellulose derivatives. It has been achieved by substitution of carboxymethyl groups (CH_2_–COOH–) bound with some of the hydroxyl groups (–OH) in cellulose macromolecule. The degree of substitution may range from 0.6 to 0.95 and is an important parameter for sol–gel transition behavior and mechanical strength. CMC has proven ideal features such as low cost, solubility in hot or cold water, non-toxicity, biocompatibility and biodegradability, and it is shown to be safe with no side effects on human health for biomedical applications (Dapia et al. 2003; Hamano et al. 1998; Salum et al. 2001). In the present work, detailed investigations were done on the effect of CS/CMC/BGP blend composition on the gelation process for potential application of an injectable product. We suggest thermosensitive CS/CMC/BGP hydrogel to solidify HCF as a new approach for effective control of spillage during the aspiration of hydatid cysts.

## Experimental

### Materials

Medium molecular weight chitosan was purchased from Sigma-Aldrich Co., Germany (deacetylation degree: 75–85 and viscosity: 0.2–0.8 Pa.s at 25 °C). Carboxymethyl cellulose with molecular weight of 250,000 and degree of substitution of 0.78 was purchased from Shazand Petrochemical Co. Arak, Iran. *β*-Glycerophosphate (G9422) with molecular weight of 316.04 g/mol was purchased from Sigma-Aldrich Co., Germany. Hydatid cyst fluid (HCF) was aspirated from hydatid cysts obtained from livers of sheep slaughtered at the local abattoir. Alamar Blue cell viability reagent was purchased from Thermo Fisher Scientific Co. Acetic acid was purchased from Merck Co. Distilled water was used throughout the experiments.

## Methods

### Preparation of thermosensitive hydrogel

**Fig. 1:**
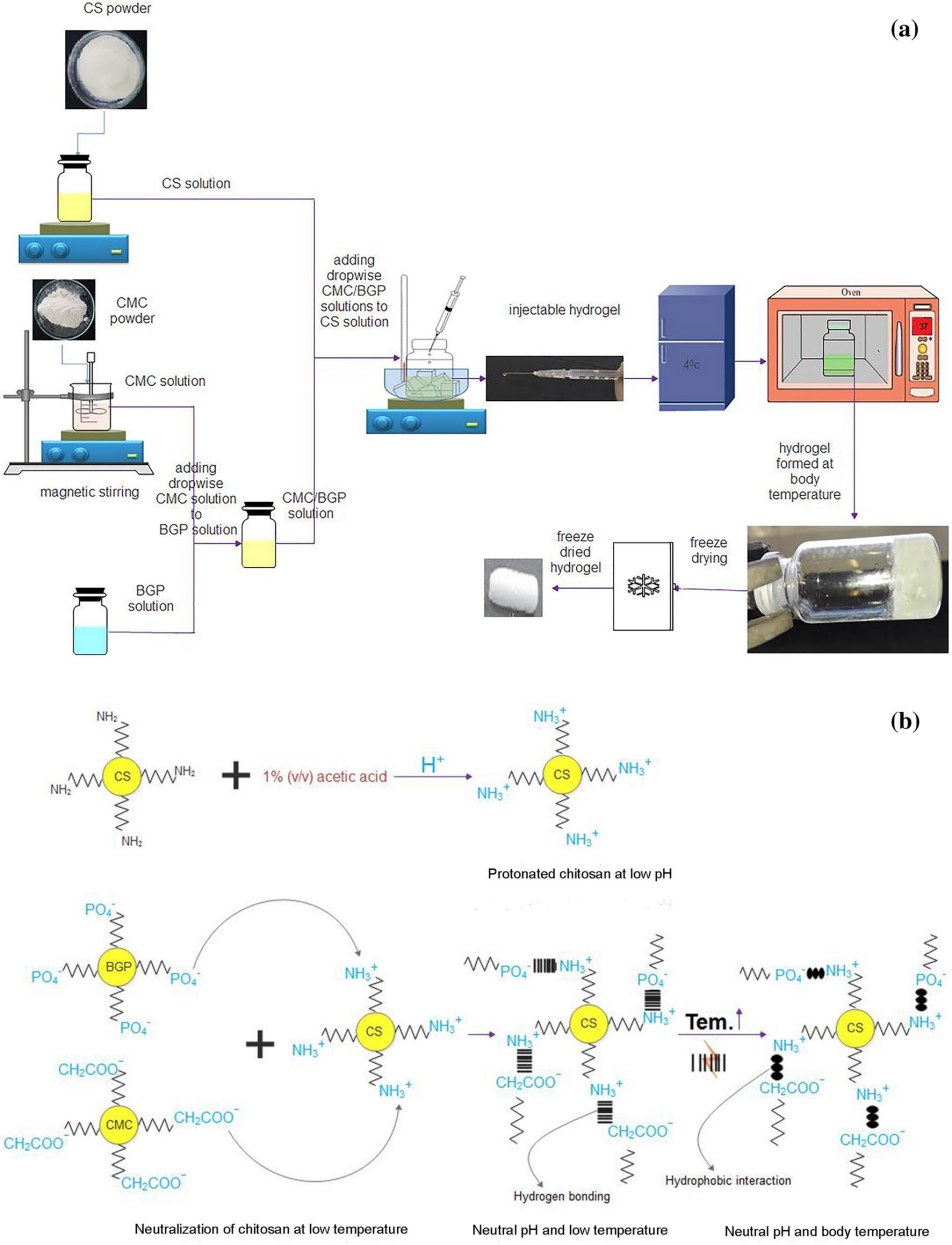
**a** The schematic of experimental procedure for synthesis of thermosensitive hydrogel. **b** The schematic of gel formation mechanism

Figure 1a shows the schematic representation of the experimental procedure. Briefly, a series of chitosan powders was dissolved in 1% (v/v) acetic acid at room temperature and stirred overnight by magnetic stirring until a clear homogeneous solution were observed and chilled in an ice bath at 4 °C for 30 min. Different CMC solutions were prepared by gradual dispersion of CMC into hot water. Full CMC dissolution was achieved by storage in a refrigerator (4 °C) for 1 day prior to use. Different BGP solutions were added to CMC solutions, mixed and cooled for 30 min. CMC/BGP solutions were added dropwise to CS solutions while cooling in an ice/water bath under magnetic stirring for 20 min. The resultant solutions were maintained at 4 °C for further studies. The final pH values were made to approach to neutralization using a pH meter (pH/mv/TEMP Meter P25, Korea). The final solutions included CS (1–2%), CMC (0.8–1.6%) and BGP (2–5.9%).

### Design of experiments

A five level with three times replications at the central point of the Design-Expert Statistical Software (version 10.0.3 Stat Ease, Inc.) using a central composite design (CCD), which is a response surface methodology (RSM), was used for the model fitting and experiment optimization of response parameters. Independent variables in this work were considered: chitosan solution (A), carboxymethyl cellulose solution (B) and β-glycerophosphate solution (C) concentrations. The pH values, gel times and water uptakes were considered as responses (dependent variables). The minimum, center and maximum levels of independent variables were characterized with − *α*, 0 and + *α*, in the order given. The ranges of variables were determined based on pre-tests. In Table 1, the range of changes in variables is presented. The number of experiments required is given by the expression: 2^k^ (2^3^ = 8; factorial point) + 2 k (2 × 3 = 6; axial points) + 3 (center points; 3 replications), where k is the number of factors (Montgomery 2012). Seventeen experimental runs with three factors and five levels and three replications were carried out at center points. ANOVA was used to analyze the statistical model for the relationship between independent variables as inputs and dependent variables as responses and their interactions based on experimental data while ignoring some outliers. The results were evaluated with various statistical parameters such as *t* value, probability (*p* value), Fisher (*f* value), degrees of freedom, correlation coefficient (*R*^2^), adjusted correlation coefficient *R*^2^ and lack of fit for model signification and lack of fit for not signification. *F* values and *p* values show that at 95% confidence level (*p* < 0.05), all of predicted models based on experimental data were significant and lack of fits were not significant.

**Table 1:** Composition of materials in the studied range

Concentration of variables	Variable levels				
	− α	− 1	0	+ 1	+ α
CS	1	1.25	1.5	1.75	2
CMC	0.8	1	1.2	1.4	1.6
BGP	2	2.9	3.8	4.7	5.6

### Scanning electron microscopy (SEM)

The prepared hydrogels were frozen at − 70 °C and freeze dried for 24 h at pressure of 2 Pa (0.02 mbar) (freeze dried to avoid damage to the porous structure without any breakdown). The samples were cut with a surgery knife and mounted on aluminum holders. Then, the samples were sputter-coated with gold and the porous morphologies were imaged at 20 kV acceleration voltage and 100 × magnification on a scanning electron microscope (VEGA3 TESCAN, Czech Republic).

### Fourier-transform infrared spectroscopy (FTIR)

FTIR spectra were examined using a Nicolet FT-IR NEXUS 670 spectrophotometer (USA) at ambient conditions. CS powder, CMC powder and freeze-dried hydrogel samples were crushed with KBr to prepare its discs for FTIR spectroscopic analysis to analyze their chemical structures at 500–4000 cm^−1^.

### Test tube inversion method

The sol–gel transition behavior of the hydrogels was examined by visual inspection, using the test tube inversion method (Wu et al. 2006). For this purpose, 5 mL of thermosensitive solutions were poured into a medicine bottle and stored at 4 °C for 12 h to eliminate the confined bubbles. For gelation, all samples were placed in a water bath at 37 °C. The samples were removed at 1 min intervals and reclined horizontally. Hydrogel is defined to be at solution phase if it displays liquid-like behavior and at a gel phase if it becomes immobile.

### Rheological measurements

Rheological experiments were carried out using an oscillatory cylindrical rheometer (MCR 301 Anton paar, Germany) to characterize the viscoelastic properties. Instruments apply oscillating stimulus strain to samples and induce oscillating response stress. The strain applied can be described by a sine function where *γ*_0_ is the maximum strain applied, *ω* is the frequency of applied strain, *t* is time and *δ* is the phase angle between response and stimulus. Strain and ″ stress can be expressed with Eqs. 1–4:

1 $$ \gamma \; = \;\gamma_{0} \;\sin \;(\omega t), $$

2 $$ \sigma \; = \;\sigma_{0} \;\cos \;(\omega t\; + \delta ), $$

3 $$ G^\prime \; = {{\sigma_{0} } \mathord{\left/ {\vphantom {{\sigma_{0} } {\gamma_{0} \;\cos \;\delta }}} \right. \kern-0pt} {\gamma_{0} \;\cos \;\delta }}, $$

4 $$ G^{\prime\prime}\; = {{\sigma_{0} } \mathord{\left/ {\vphantom {{\sigma_{0} } {\gamma_{0} \;\sin \;\delta }}} \right. \kern-0pt} {\gamma_{0} \;\sin \;\delta }}. $$

Dynamic viscoelastic parameters such as elastic modulus (*G*′) and viscous modulus (G″) and tan *δ* were measured as a function of time, temperature and frequency. For the gelation of the CS/MC/BGP system, a linear viscoelastic region was chosen, where *G*′ and G″ were independent of strain amplitude. So, measurements were carried out at a strain amplitude of 20%.

### Water uptake studies

Freeze-dried hydrogel samples were weighed carefully and immersed in excess distilled water at 37 °C and neutral pH. The swollen hydrogels were removed with tweezer at 30 min time intervals, wiped out with a filter paper on its surface, and then weighed and the process was repeated until constant weights were obtained. Equilibrium swelling was measured by Eq. 5 (Tang et al. 2007).

5 $$ {\text{Equilibrium}}\;{\text{swelling}}\,\,({\text{ES}})\; = \;\frac{{W_{2} \; - W_{1} }}{{W_{1} }}, $$

where *W*_1_ is the weight of dry hydrogels and *W*_2_ is the weight of swollen hydrogels.

### Alamar Blue assay

The Alamar Blue assay is a colorimetric/fluorometric growth indicator in the response to the metabolic activity. It was carried out to quantitatively evaluate the in vitro cytotoxicity and viability of fibroblast human cell lines. Alamar Blue is a fast, non-toxic, simple, water-soluble, highly sensitive, cost-effective reagent and is considered a preferred alternative reagent compared to MTT. It is an oxidation–reduction indicator, which is indigo blue in oxidation state and after reduction by cell line, bacteria and fungi its color changes to pink. So if the cell line, bacteria and fungi grow in the cultured media containing Alamar Blue, cultured media color will change from blue to pink. Alamar Blue cytotoxicity assay was used to evaluate the half maximal inhibitory concentration (IC50) of normal fibroblast human cell lines. Cytotoxicity evaluation was specified with IC50 values (Al-Nasiry et al. 2007).

## Results and discussion

### Gel formation mechanism

The mechanism of thermosensitive hydrogels has been previously studied (Li et al. 2014; Modrzejewska et al. 2014; Tang et al. 2010). The schematic of the gel formation mechanism is presented in Fig. 1b. Chitosan is a pH-dependent cationic natural polymer which is not dissolved in aqueous solutions commonly. The protonation of free amino groups of chitosan (–NH_2_) in dilute acetic acid solution can dissolve chitosan chains. The solubility of chitosan solutions (pH values below 6.2) is not desirable in physiological pH range due to lack of thermal sensitivity that limits its use for biomedical applications. The addition of a weak base like CMC/BGP with anionic head reduces the electrostatic repulsion due to protonated free amino groups of chitosan chains and maintains chitosan in solution state at low temperature and physiological pH range without any precipitation. CMC/BGP solution as the weak base can absorb protonated free amino groups (NH_3_^+^) and gradually increase the pH ,which leads to form hydrogen bonding and chains associations. As a result, a gel-like structure forms at desirable physiological condition. By adding CMC/BGP solution at low temperature, weak interactions between the molecules of chitosan, CMC, BGP and water via hydrogen bonds lead to the dissolution of CMC/BGP solutions in chitosan chains due to the increment of hydrophilicity of the solutions. This can promote the pH values close to the neutral state at low temperature and inhibit association of chitosan chains. Under this condition, the solution in the physiological pH range is injectable. Increasing temperature can disrupt the hydrogen bonding interaction between chains and accelerate their mobility. So, water molecules surrounding the polymers are dissipated and dewatered hydrophobic chains associate with each other. Therefore, hydrophobic interactions are proposed to be the main driving force in gelation of chitosan in the presence of CMC/BGP at body temperatures. Adding BGP, with high anion charge density, results in greater interaction with water molecules which reduces intermolecular hydrogen bonding between the water molecules and polymer chains. It can permit greater hydrophobic interaction between CMC and CS chains, leading to more rapid onset of gelation near the body temperature (Tang et al. 2010).

### SEM observation

**Fig. 2:**
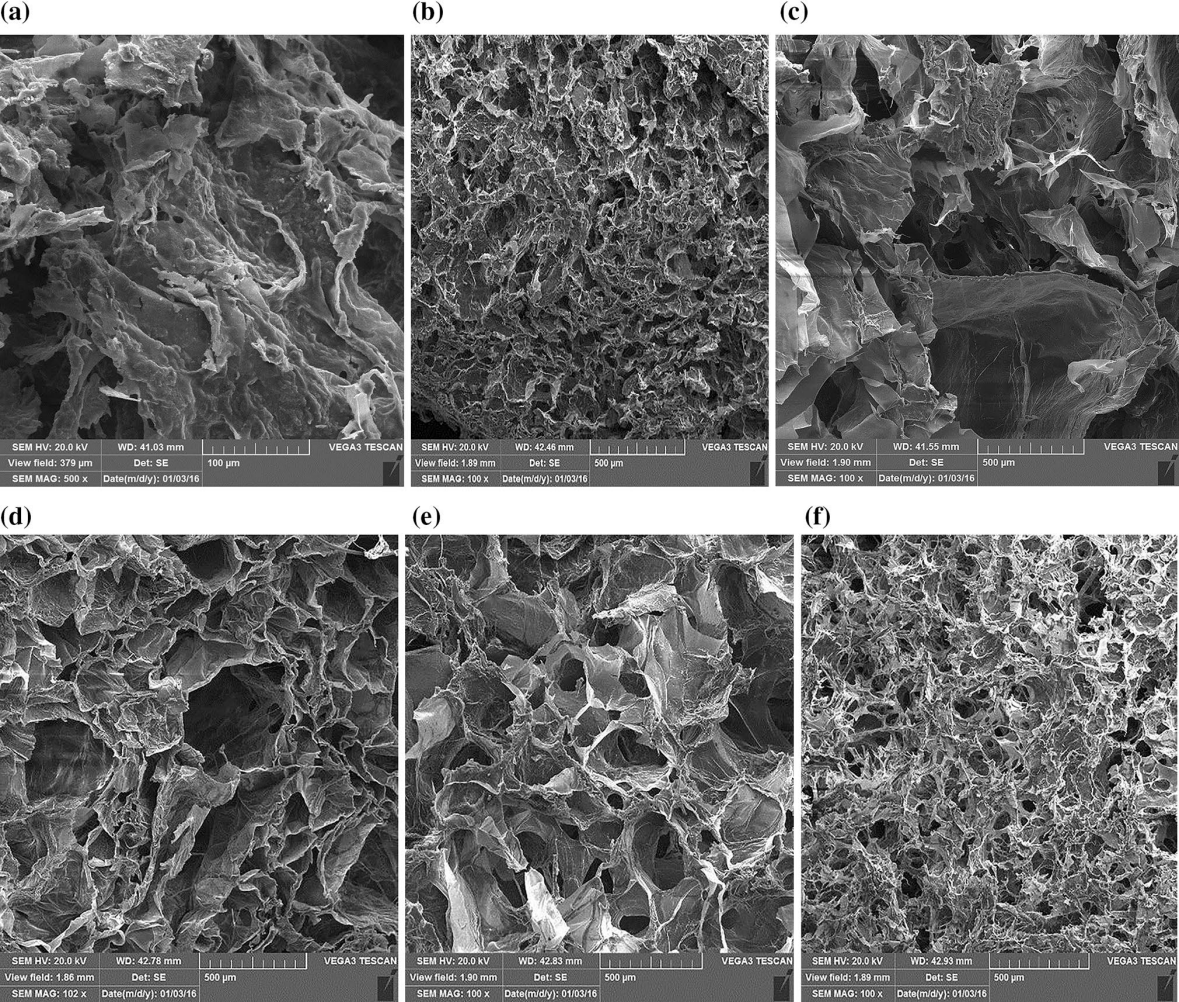
SEM images of fracture section of different contents of CS/CMC/BGP blend hydrogel, **a** CS(1.25%)/CMC(1%)/BGP(4.7%), **b** CS(1.75%)/CMC(1.4%)/BGP(4.7%), **c** CS(1.75%)/CMC(1%)/BGP(4.7%), **d** CS(1.25%)/CMC(1.4%)/BGP(2.9%), **e** CS(1.75%)/CMC(1.4%)/BGP(2.9%), **f** CS(1.75%)/CMC(1%)/BGP(2.9%)

The microstructure of the freeze-dried CS/CMC/BGP blend hydrogels was analyzed using scanning electron microscopy (SEM). Figure 2a–f shows SEM images of the fracture section of hydrogels and the effect of different CS, CMC and BGP concentrations on the morphology of the blend hydrogels. SEM micrographs showed that the pore morphology in different samples changed from nanoporous to microporous, which clearly depended on the CS, CMC and BGP contents. The results demonstrated that with a constant content of chitosan concentration, the pore size became smaller and hydrogels had denser structure with increase of CMC and BGP concentrations due to the ionic interactions between CH2COO^−^ and PO4^−^ with NH_3_^+^ and physical chain interactions between CS and CMC and vice versa. Therefore, CS (1.75%)/CMC (1.4%)/BGP (4.7%) hydrogel with higher BGP content had a denser structure with smaller pores in comparison with CS (1.75%)/CMC (1.4%)/BGP (2.9%). Also with the rise in CS concentration (comparing the Fig. 2a with b or d with e), the number of CS molecules within the gel structure increased leading to shorter interparticle distance and, hence, hydrogels with a uniform microporous network were observed. In addition, the CS (1.75%)/CMC (1%)/BGP (4.7%) sample did not have a uniform micropore structure and showed a poor pore interconnectivity, while in CS (1.75%)/CMC (1.4%)/BGP (4.7%) and CS (1.75%)/CMC (1.4%)/BGP (2.9%) hydrogels, a uniform network with good interconnectivity between pores was observed. This indicated a good interaction between the two polymers in the ratio of CS/CMC = 1.75/1.4.

### FTIR analysis

**Fig. 3:**
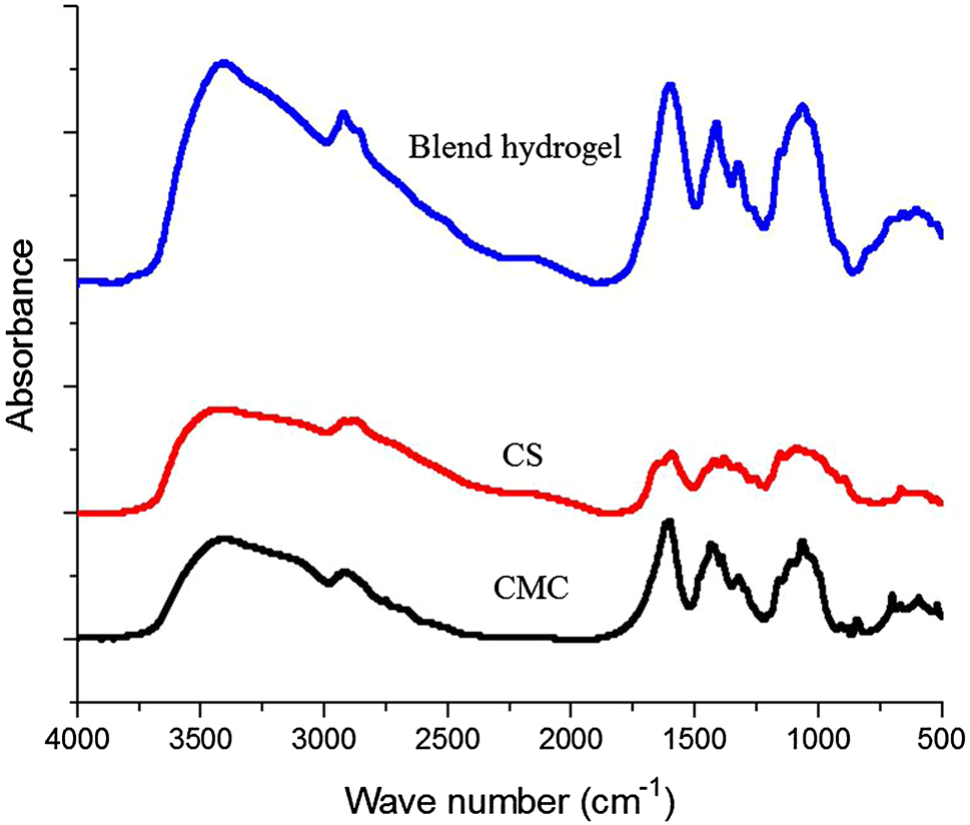
FTIR spectra of freeze-dried blend hydrogel plus CS and CMC

The FTIR spectra of freeze-dried CS/CMC/BGP hydrogel samples (pure CS and CMC) are shown in Fig. 3. For pure chitosan, absorption peaks are observed at ∼ 3400–3500 cm^−1^ (O–H stretching of intermolecular hydrogen bonding), ∼ 2900 cm^−1^ (stretching vibrations in aliphatic groups –CH_2_ and CH_3_), ∼ 1650 cm^−1^ (C=O stretch of the amide bond, amide I), ∼ 1605 cm^−1^ (–NH_2_ bending), ∼ 1590 cm^−1^ (C=O stretching vibrations of amide II), ∼ 1420 cm^−1^ (oscillation characteristics of C–H bending of CH_2_ groups), ∼ 1380 cm^−1^ (C–N stretching in secondary amide, amide III), ∼ 1250 cm^−1^ (C–O stretching of ring ether) and ∼ 1090 cm^−1^ (C–O symmetric stretching of primary alcohol). The amino group has a characteristic absorption peak at the ∼ 3400–3500 cm^−1^ region (often masked by a broad OH absorption). For pure CMC, absorption peaks are observed at ∼ 3400 cm^−1^ (OH stretching vibration), ∼ 2900 cm^−1^ (C–H stretching in CH_2_ and CH_3_), ∼ 1580 cm^−1^ (symmetric modes of stretching vibration of carboxylic groups) ∼ 1430 cm^−1^ (C–H bending in, CH_2_), ∼ 1400 cm^−1^ (asymmetric modes of stretching vibration of carboxylic groups) ∼ 1250 cm^−1^ (attributed to the C–O stretching) and ∼ 1060 cm^−1^(asymmetric stretching vibration modes of ether bonds). These observed peaks are in accordance with previous reports. The FTIR spectra of freeze-dried blend hydrogel had no additional absorbance peaks compared to those of CS and CMC. This suggests that there is no direct chemical interaction between the components, i.e., no introduction of additional chemical functionality to account for gelation. Therefore, the gelation process is a result of the physical interaction phenomena (i.e., ionic/hydrophobic interactions) between the CS and CMC chains (Tang et al. 2007, 2010).

### Water uptake analysis

**Fig. 4:**
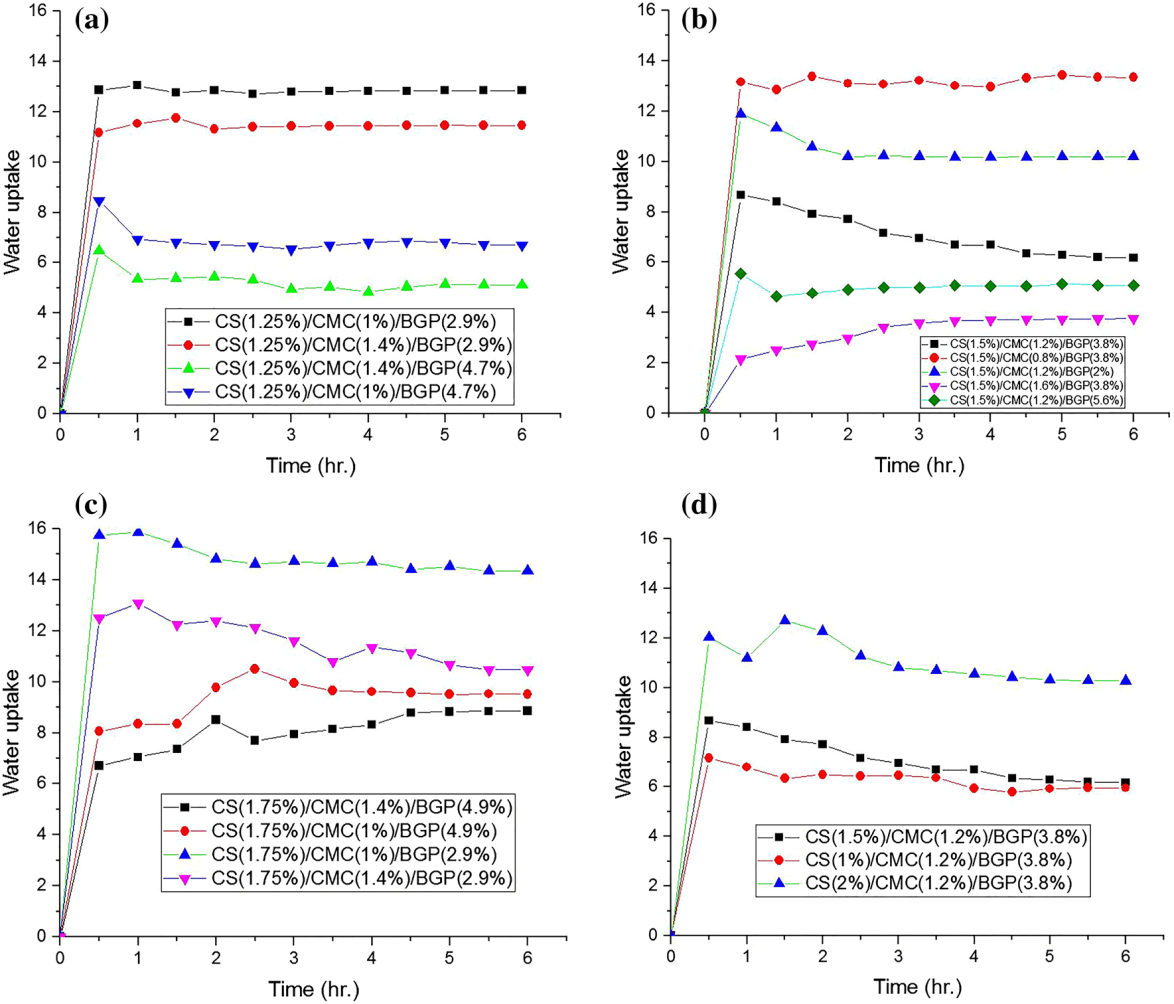
Water uptake of CS/CMC/BGP blend hydrogels in distilled water at 37 °C. **a**–**c** Constant CS contents and different CMC and BGP contents, **d** constant CMC and BGP contents and different CS contents

The results of the experimental curves of water uptake for CS/CMC/BGP hydrogels are shown in Fig. 4. These results show that a large amount of fluids was absorbed by hydrogels during the initial 30 min and appreciable water uptake with a good strength was obtained. This result demonstrated that the swelling ratio was decreased with increase of CMC and BGP concentrations when the chitosan concentration was constant. The reason is relatively simple. A higher concentration of CMC and BGP leads to increased entanglements between the chains and thus the hydrogel structure becomes denser and the hydrogel pore sizes decrease. For example, CS (1.75%)/CMC (1.4%)/BGP (2.9%) had higher equilibrium water uptake (10.48) as compared to CS (1.75%)/CMC (1.4%)/BGP (4.9%) with equilibrium water uptake of 8.85. In addition, CS (1.25%)/CMC (1.4%)/BGP (4.7%) had lower water uptake than CS (1.25%)/CMC (1.0%)/BGP (4.7%) due to a higher concentration of CMC at the same content of CS and BGP (Fig. 4a).

### Rheological analysis

**Fig. 5:**
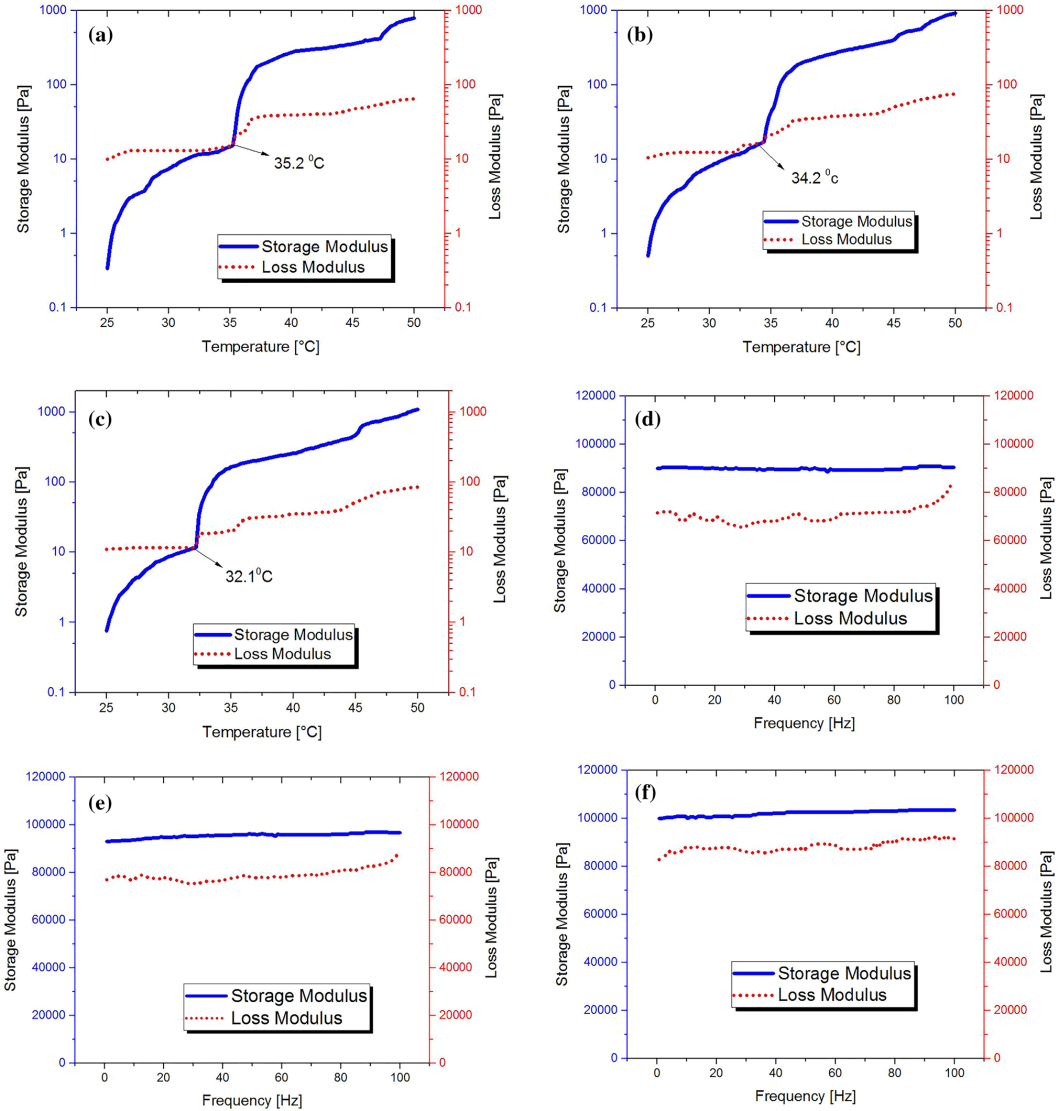
Rheological properties of blend hydrogel at constant CS and CMC content and different BGP contents. Temperature dependence of storage modulus *G*’ and loss modulus *G*’’ of **a** CS (1.75%)/CMC (1.4%)/BGP (2.9%), **b** CS (1.75%)/CMC (1.4%)/BGP (3.8%), **c** CS (1.75%)/CMC (1.4%)/BGP (4.7%). Frequency dependence of storage modulus *G*’ and loss modulus *G*’’ of **d** CS (1.75%)/CMC (1.4%)/BGP (2.9%), **e** CS (1.75%)/CMC (1.4%)/BGP (3.8%), **f** CS (1.75%)/CMC (1.4%)/BGP (4.7%)

Dynamic mechanical analysis tests were performed as a function of temperature and frequency. For temperature sweep test, aliquots of test samples at a constant frequency of 1 rad/s and constant oscillation amplitude 20% were loaded into the rheometer and allowed to equilibrate (5 min). The temperature was increased from 25 to 50 °C at a rate of 2 °C/min. The storage modulus (*G*′) and the loss modulus (*G*″) values were plotted versus temperature in Fig. 5a–c. The storage modulus expresses the elastic part of the viscoelastic behavior, which is low at solution state but increases dramatically at the gelation temperature. The region in which *G*′ is lower than *G*″ shows that the viscous behavior is dominant, and where *G*′ is higher than *G*″, the elastic behavior is dominant as a result of increase of hydrophobic interaction. The *G*′, *G*″ crossover point is a measure of the gelation temperature and is usually defined as the sol/gel transition temperature (Tang et al. 2010). According to Fig. 5a–c, the gel temperatures for CS (1.75%)/CMC (1.4%)/BGP (2.9%), CS (1.75%)/CMC (1.4%)/BGP (3.8%) and CS (1.75%)/CMC (1.4%)/BGP(4.7%) solutions are 35.2, 34.2 and 32.1 °C, respectively. The results show that the increase of final pH solution shifts the gelation temperature to lower value. This is due to the fact that the increase of pH leads to more association of CS and CMC chains and hydrogel formation takes place at lower temperature. In the frequency sweep test, measurements were made over a range of oscillation frequencies of 0.1–100 Hz at constant oscillation amplitude 20% and temperature 37 °C. Figure 5d–f shows the change of storage modulus (*G*′) and the loss modulus (*G*″) versus frequency. As can be seen, the storage modulus *G*′ is almost independent of frequency and greater than loss modulus over the whole frequency range. This solid-like structure demonstrates that the body temperature is higher than the gelation temperature. At constant CS and CMC contents, the increase in BGP led to increase of the storage modulus (*G*′). As expected, higher BGP salt concentration with anion charge results in greater interaction with water molecules, thereby reducing intermolecular hydrogen bonding between water molecules and CS/CMC chains, which permits greater hydrophobic interaction between the chains to produce a stronger hydrophobically cross-linked network.

### Experiment design analysis

The design matrix and their ranges of independent variables, which are CS, CMC and BGP concentrations, and responses, which are gel time, pH values and equilibrium water uptake, respectively, are presented in Table 2. The pH values, gelation times and equilibrium water uptakes ranged between about 6.51 and 7.15, 41 and 92 min and 3.76 and 14.34, respectively. CCD was applied for the development of the polynomial regression equations which were quadratic expressions for gel times and equilibrium water uptakes and 2Fl for pH values as recommended by the software. In regression, the goodness of fit between the predicted and experimental data was shown by *R*^2^ values in every model that were 0.9769 for pH values, 0.9983 for gelation times and 0.9952 for equilibrium water uptakes, which were within the desirability range. The *R*^2^ values were in conceivable agreement with the adjusted *R*^2^ value and were 0.9571, 0.9944 and 0.9845 for pH value, gelation time and equilibrium water uptake, respectively (difference between *R*^2^ values and adjusted *R*^2^ value for every response were less than 0.2). The final empirical models for pH values, gelation times and equilibrium water uptakes are given by Eqs. (6) and (7) and (8), respectively. Positive and negative signs before the terms designated the synergetic and antagonistic effects of the corresponding variables. For example in empirical models of equilibrium water uptake, the negative sign before CMC and BGP content is due to the fact that higher contents lead to lower equilibrium water uptake individually. The center point of CCD with three times replications is used to calculate the experimental error and reproducibility of the data. The sample standard deviations for pH, water uptake and gelation time are 0.025, 0.23 and 1.53 min, respectively.

**Table 2:** Experimental design matrix using central composite design

Independent variables (% wt/v)				Responses		
Run	CS	CMC	BGP	pH gel time (min)	Water uptake	
1	1.25	1.40	4.70	7.08	45.00	5.11
2	1.25	1.40	2.90	6.66	77.00	11.44
3	1.50	1.60	3.80	6.87	65.00	3.76
4	1.50	1.20	2.00	6.63	80.00	10.19
5	2.00	1.20	3.80	6.88	65.00	10.27
6	1.50	1.20	5.60	7.00	51.00	5.07
7	1.50	1.20	3.80	6.76	72.00	6.68
8	1.50	1.20	3.80	6.79	71.00	6.41
9	1.50	0.80	3.80	6.70	76.00	13.34
10	1.50	1.20	3.80	6.74	74.00	6.17
11	1.00	1.20	3.80	6.58	87.00	5.95
12	1.25	1.00	4.70	6.92	54.00	6.70
13	1.25	1.00	2.90	6.51	92.00	12.83
14	1.75	1.00	2.90	6.59	82.00	14.34
15	1.75	1.40	4.70	7.15	41.00	8.85
16	1.75	1.40	2.90	7.03	50.00	10.48
17	1.75	1.00	4.70	7.11	44.00	9.51

### Statistical analysis

**Fig. 6:**
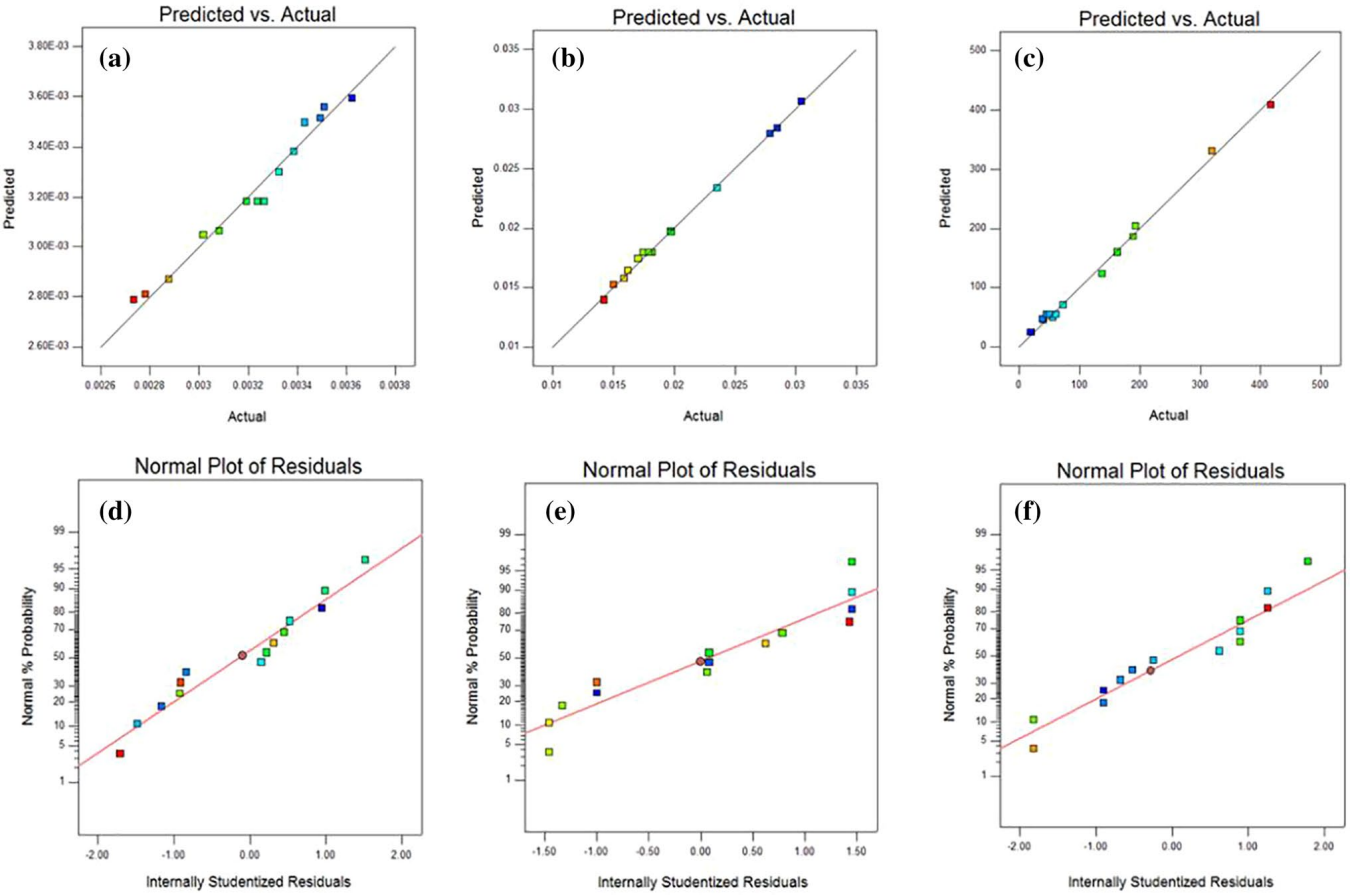
Relationship between predicted and experimental data for **a** the pH values, **b** gel times and **c** equilibrium water uptakes Comparison between normal probability and internally studentized residuals for **d** the pH values, **e** gel times and **f** equilibrium water uptakes

The suitability of the models was evaluated with analysis of variance (ANOVA) to study the effects of the variables and their interactions. The analysis of variance (ANOVA) is given in Table 3 for the 2Fl model for pH values and second-order quadratic models for gelation time and equilibrium water uptake. It shows the sum of squares and mean square of each factor, *F* value as well as Prob > *F* values. Table 4 shows the sum of the squares of each of the variation sources. The model terms with value of Prob > *F* less than 0.05 are considered as significant. From the obtained results for pH value, water uptake, gel time the model *F* value and Prob > *F* was 49.39 and < 0.0001, 103.22 and 0.0002, 255.59 and < 0.0001, respectively. Results show that for pH value, *AC* term, water uptake, *AB* and *AC* terms and gelation time, *A*^2^ and *B*^2^ terms were insignificant and hence excluded from the final equations. The lack of fit values for all models was not significant, showing that the fitted models are significant. From the statistical results obtained, it can be seen that the models were suitable in predicting the pH value, gelation time and equilibrium water uptake within the range of the studied variables. Additionally, Fig. 6a–c shows the predicted values versus the experimental values for the pH value, gelation time and equilibrium water uptake, respectively. It is observed that models successfully predict the relation between initial concentrations to the responses. The values of coefficient of variation (CV) were 1.80, 1.98 and 9.99 for the pH value, gelation time and equilibrium water uptake, respectively. the CV value declares the percentage ratio of standard deviation to the mean value for each variable. The lower CV value led to higher reproducibility. The normal probability versus internally studentized residuals is shown in Fig. 6d–f, which reveals a normal distribution of data for all responses as an important topic in statistical description. Adeq Precision is a measurement of the signal to noise ratio. A ratio more than 4 is eligible. The results for the pH value, gelation time and equilibrium water uptake were 19.788, 49.473 and 33.231, respectively which exhibited a suitable signal.

**Table 3:** ANOVA and lack-of-fit test for the response 2Fl model for the pH values

Source	pH					
	Sum of squares	*df*	Mean square	*F* value	*P* value Prob > *F*	Remark
Model	9.87E−7	6	1.65E−7	49.39	< 0.0001	Significant
A-CS	3.48E−7	1	3.48E−7	104.49	< 0.0001	
B-CMC	4.67E−8	1	4.67E−8	14.02	0.0072	
C-BGP	2.19E−7	1	2.19E−7	65.76	< 0.0001	
AB	6.72E−7	1	6.72E−8	20.18	0.0028	
AC	9.24E−9	1	9.24E−9	2.78	0.1396	
BC	1.41E−7	1	1.41E−7	42.29	0.0003	
Residual	2.33E−8	7	3.33E−9			
Lack of fit	2.07E−8	5	4.14E−9	3.19	0.2556	Not significant
Pure error	2.59E−9	2	1.29E−9			
Cor total	1.01E−6	13				

Std. dev.	Mean	C.V. %	Press	*R* ^2^	Adj. *R*^2^	Pred. *R*^2^	Adeq. Precision
5.77E−5	3.21E−3	1.80	1.16E−7	0.9769	0.9571	0.8849	19.788

**Table 4:** ANOVA and lack-of-fit test for the response quadratic model for gel times and equilibrium water uptakes

Source	Gel time (min)					Water uptake					
	Sum of squares	*df*	Mean square	*F* value	*F* value Prob > *F*	Sum of squares	*df*	Mean square	*F* value	*F* value Prob > *F*	Remark
Model	3.67E−4	9	4.08E−5	255.59	< 0.0001	1.07E+5	9	11893.82	103.22	0.0002	Significant
A-CS	1.55E−5	1	1.55E−5	97.21	0.0006	11429.65	1	11429.65	99.19	0.0006	
B-CMC	3.68E−6	1	3.68E−6	23.08	0.0086	44726.21	1	44726.21	388.16	< 0.0001	
C-BGP	2.89E−4	1	2.89E−4	1807.90	< 0.0001	9386.43	1	9386.43	81.46	0.0008	
AB	1.52E−6	1	1.52E−6	9.55	0.0366	11.83	1	11.83	0.10	0.7650	
AC	3.03E−6	1	3.03E−6	18.98	0.0121	39.62	1	39.62	0.34	0.5891	
BC	6.21E−6	1	6.21E−6	38.85	0.0034	23450.12	1	23450.12	203.52	0.0001	
A2	2.52E−7	1	2.52E−7	1.58	0.2775	2560.61	1	2560.61	22.22	0.0092	
B2	3.77E−7	1	3.77E−7	2.36	0.1993	10473.81	1	10473.81	90.90	0.0007	
C2	8.83E−5	1	8.83E−5	522.88	< 0.0001	1717.49	1	1717.49	14.91	0.0181	
Residual	6.39E−7	4	1.59E−7			460.90	4	115.22			
Lack of fit	3.90E−7	2	1.95E−7	1.57	0.3891	413.20	2	206.60	8.66	0.1135	Not significant
Pure error	2.48E−7	2	1.24E−7			47.69	2	23.85			
Cor total	3.68E−4	13				1.07E+5	13				

Source	Std. dev.	Mean	C.V. %	Press	R^2^	Adj. R^2^	Pred. R^2^	Adeq. Precision
Gel time	3.99E−4	0.020	1.98	2.34E−5	0.9983	0.9944	0.9364	49.473
Water up take	10.73	107.47	9.99	13175.55	0.9957	0.9861	0.8774	33.231

6 $$ ({\text{pH}})^{ - 3} = \,4.38{\text{E}} - 3 + 2.51{\text{E}} - 3*A - 3.55{\text{E}} - 4*B - 9.48{\text{E}} - 4*C - 2.15{\text{E}} - 3*A*\;B + 8.67{\text{E}} - 4*\;B*\;C, $$

7 $$ ({\text{Gel time}})^{ - 0.94} = 0.06 + 0.01*\;A - 0.01*B - 0.03*C - 0.01*\;A*\;B + 3.53{\text{E}} - 3*\;A*\;C + 6.38{\text{E}} - 3*\;B*\;C + 3.64*C^{2} , $$

8 $$ ({\text{Water}}\;{\text{uptake}})^{2.17} = \,4033.25 - 441.10*A - 3785.74*B - 609.49*C + 392.04*B*C + 184.55*A^{2} + 803.5*B^{2} + 11.66*C^{2} , $$

where *A*, *B* and *C* are chitosan, carboxymethylcellulose and β-glycerol phosphate contents.

### Effects of individual variables and their interactions

**Fig. 7:**
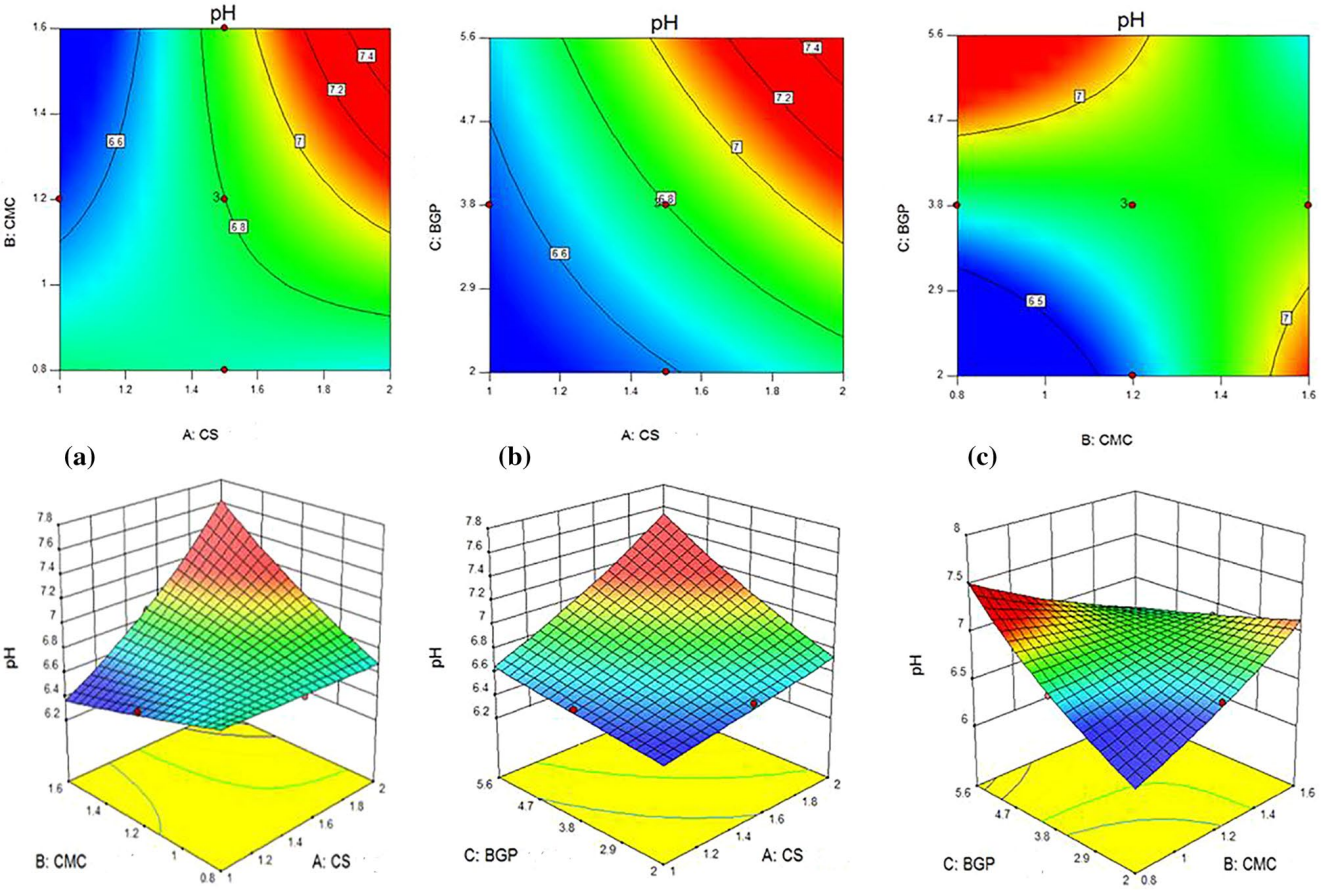

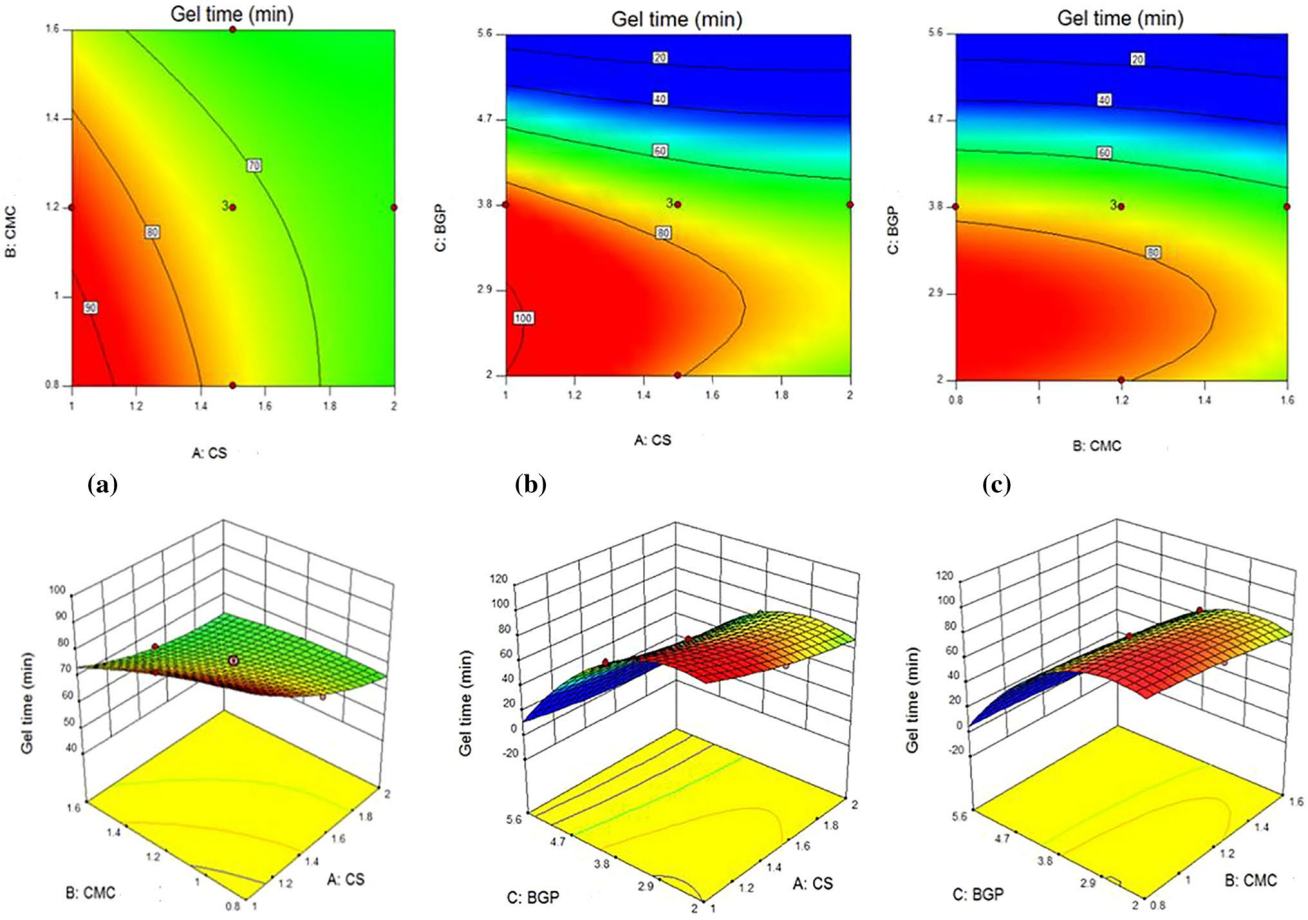

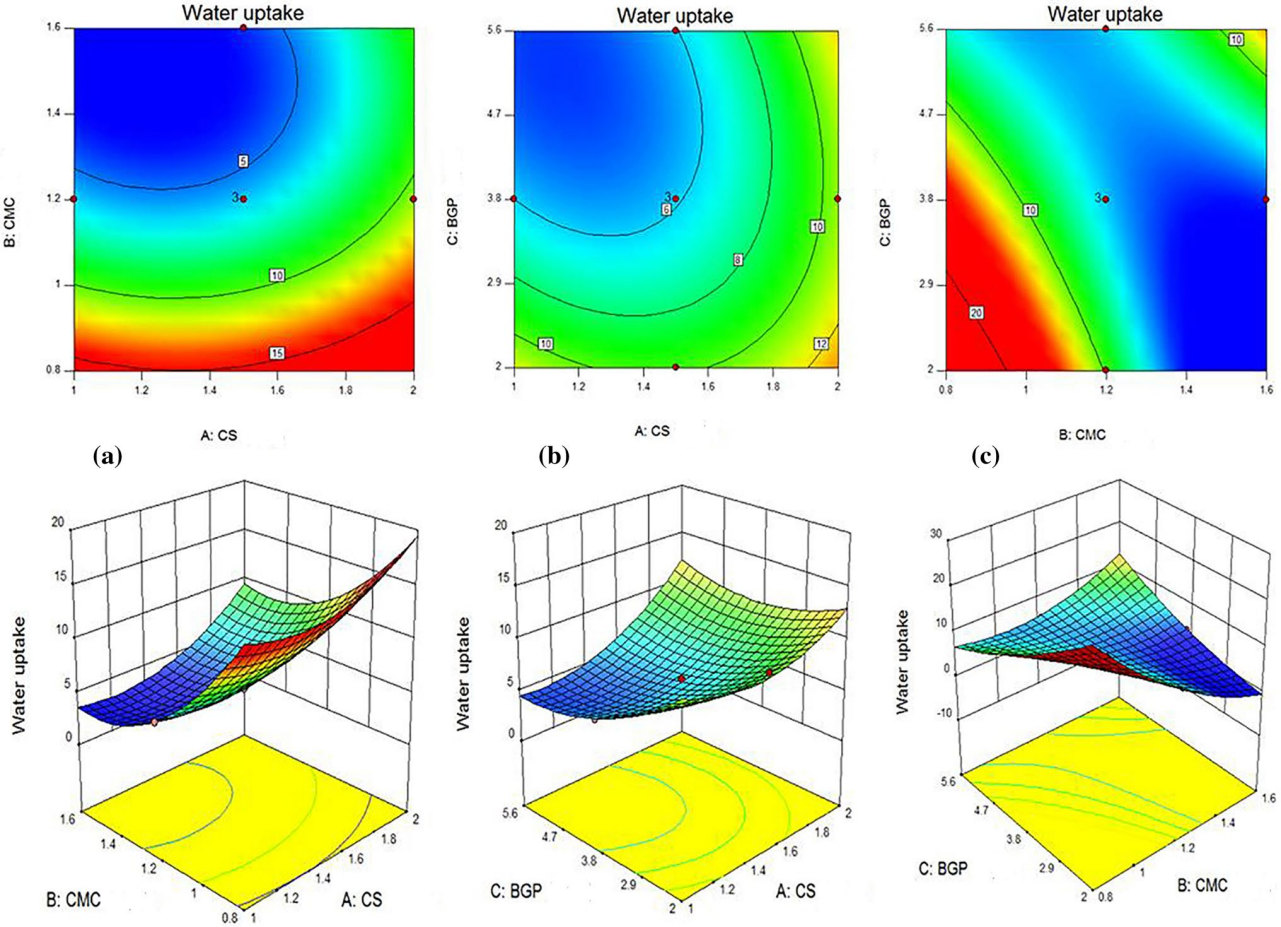
1. 2D and 3D images of combined effect of (a) CS and CMC contents, (b) CS and BGP contents and (c) BGP and CMC contents on the gel time. 2. 2D and 3D images of the combined effect of (a) CS and CMC contents, (b) CS and BGP contents and (c) BGP and CMC contents on water uptake. 3 2D and 3D images of the combined effect of (a) CS and CMC contents, (b) CS and BGP contents and (c) BGP and CMC contents on the pH value

Tables 3 and 4 show the individual effect of CS, CMC and BGP concentrations on pH values, gelation times and equilibrium water uptakes. It can be seen that for the pH values, *F* values of CS, CMC and BGP contents were 104.49, 14.02 and 65.76, respectively. It can be deducted that the pH values is more sensitive to CS solution than BGP and CMC solutions. For gelation times, *F* values of CS, CMC and BGP solutions were 97.21, 23.08 and 1807.90, respectively, and BGP content had more superior effect than the two parameters for this response. For equilibrium water uptake, the individual effects of CS solution with *F* value of 99.19, CMC solution with *F* value of 388.16, and BGP solution with *F* value of 81.46 concentrations show that the CMC solution had higher influence on this response. Figure 7 (1–3) shows the 2D contours and 3D response surface plots as a function of two parameters (by maintaining the third parameter at a constant level) which are useful for visual analysis of interaction and the combined effects of every two independent variables on responses. Nine 2D contours and 3D plots were designed for the responses.

Figure 7-1(a) shows the combined effect of CS and CMC contents on the gelation time at a constant content of BGP. As can be seen, the gelation time was decreased with the increase of both CMC and CS content in hydrogels. This decrease is because of the balanced charge between polymer chains and BGP in the experimental range due to the production of a complex network by multiple interactions (ionic, hydrophobic and hydrogen bonding, as discussed before in Sect. “Preparation of thermosensitive hydrogel”).

Figure 7-1(b) depicts the combined effect of CS solution and BGP solution on the gelation time at a constant content of the CMC solution. The gelation time was decreased with the increase in CS content with the above-mentioned mechanism, but for the second variable, at first, it was nearly increased and afterward decreased with the BGP content. It seems that BGP salt at lower concentration cannot disrupt inter-macromolecular hydrogen bonding, and shield electrostatic/ionic repulsion/attraction effects. On the contrary, the salt ions are attracted by the water molecules and intensify the cage-like shielding effect around the CS chains; hence an initial increase in gelation time can be observed. However, as the BGP concentration increases, they can attract the water molecules away from the CS chains, resulting in the direct ionic interaction between BGP and protonated CS to produce the gel with the lower time in the range of body temperature.

Figure 7-1(c) shows the combined effect of BGP and CMC content on the gelation time at a constant content of CS. Resultant curve depicts that at low BGP concentration, its increase leads to longer gelling time. However, at higher BGP content, this trend is opposite. The possible mechanism for this behavior is discussed above for Fig. 7-1(b). As shown in Fig. 7-1(c), for CS/CMC/BGP hydrogels, the increase of CMC content leads to a decreased gelation time. Its reason may be due to more association of CS and CMC chains which reduces the gelling time.

Figure 7-2(a) presents the combined effect of CS solution and CMC solution on equilibrium water uptakes at a constant content of BGP solution. It is observed that by increasing both CMC and CS content, firstly the equilibrium water uptake is decreased and then increased. But the sensitivity of this physical property to CMC content is more than that of CS content in this experimental range.

Figure 7-2(b) shows the combined effect of CS and BGP contents on equilibrium water uptake at a constant content of CMC solution. As can be seen, increasing CS content and decreasing BGP content lead to increased equilibrium water uptake, because based on SEM images, increasing CS content produces a uniform microporous hydrogel with appropriate pore connectivity and more water uptake. According to SEM results (Fig. 2), CS/CMC/GP hydrogels show more compact morphology and smaller holes at higher BGP content that results in lower water uptake.

Figure 7-2(c) shows the combined effect of BGP solution and CMC solution on equilibrium water uptake at a constant content of CS solution. As shown, the trend of water uptake with CMC and BGP change at the lower and upper limits of each variable is not the same and that complicates the mechanism.

Figures 7-3(a, b, c) presents the effects of CS, CMC and BGP content on the pH value. Overall, the results reveal that the pH value is increased with the increase of CMC and BGP content and almost unchanged with CS content. Higher interaction of CMC with carboxymethyl ether group in the sodium salt form (–O–CH_2_–COO^−^Na^+^) and basic nature of BGP decrease the concentration of H^+^ ions, which in turn result in pH increase of the polymer solution.

### Perturbation plot

**Fig. 8:**
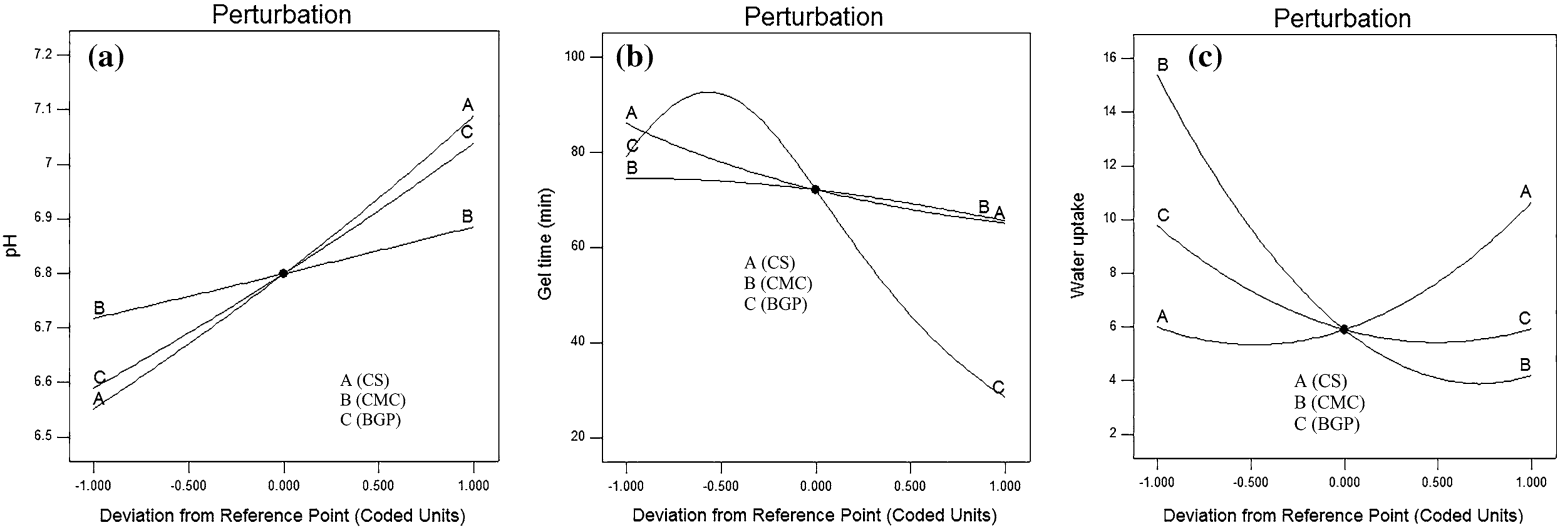
Overlay plots of perturbation for **a** pH values, **b** gel time and **c** equilibrium water uptake

The perturbation plot was applied to study the sensitivity of three parameters simultaneously on the pH value, gel time and equilibrium water uptake. As can be seen in Fig. 8a, b, the effectiveness of components for the equilibrium water uptake is in the order CMC > CS > BGP and for the gel time in the order BGP > CS > CMC. As shown in Fig. 8c, the effect of each variable on the pH value is as follows: CS > BGP > CMC. These results are in accordance with f values of ANOVA analysis.

### pH sensitivity of hydrogel

**Fig. 9:**
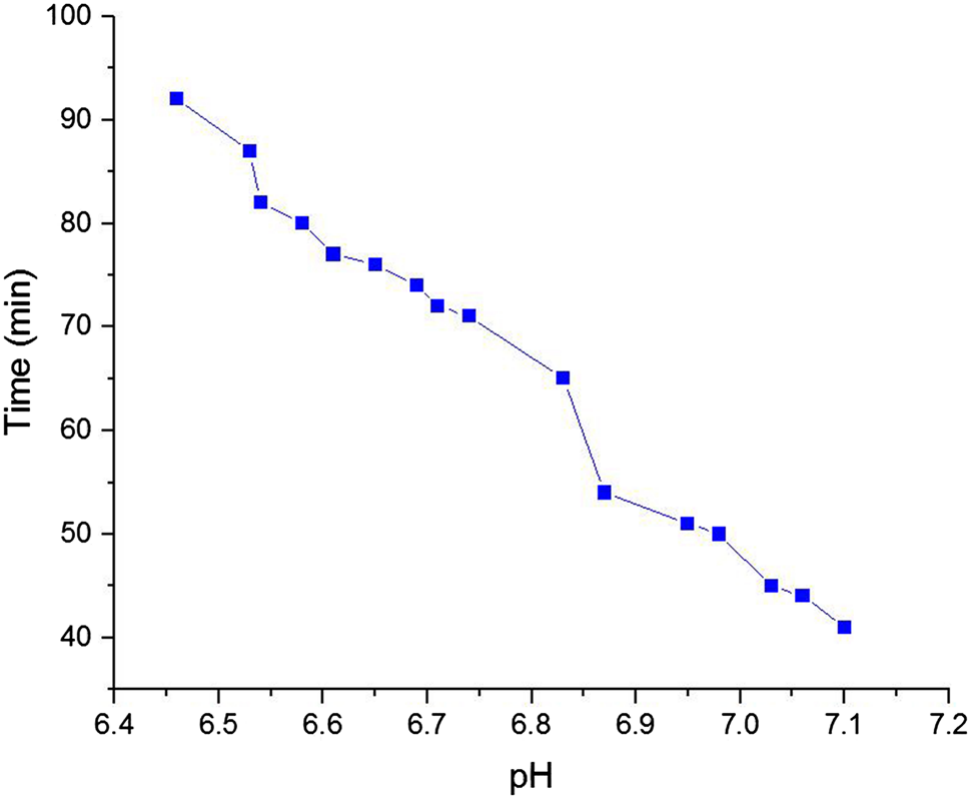
pH sensitivity of hydrogel

The relationship between pH values versus gelation times was studied. It can be obviously seen (Fig. 9) that the increase of pH value decreases the gelation time. Its reason is due to the fact that the increase in pH value leads to more association between polymer chains and thus neutralization and subsequently gelation will take place in a shorter time at body temperature.

### In vitro solidification of HCF

**Fig. 10:**
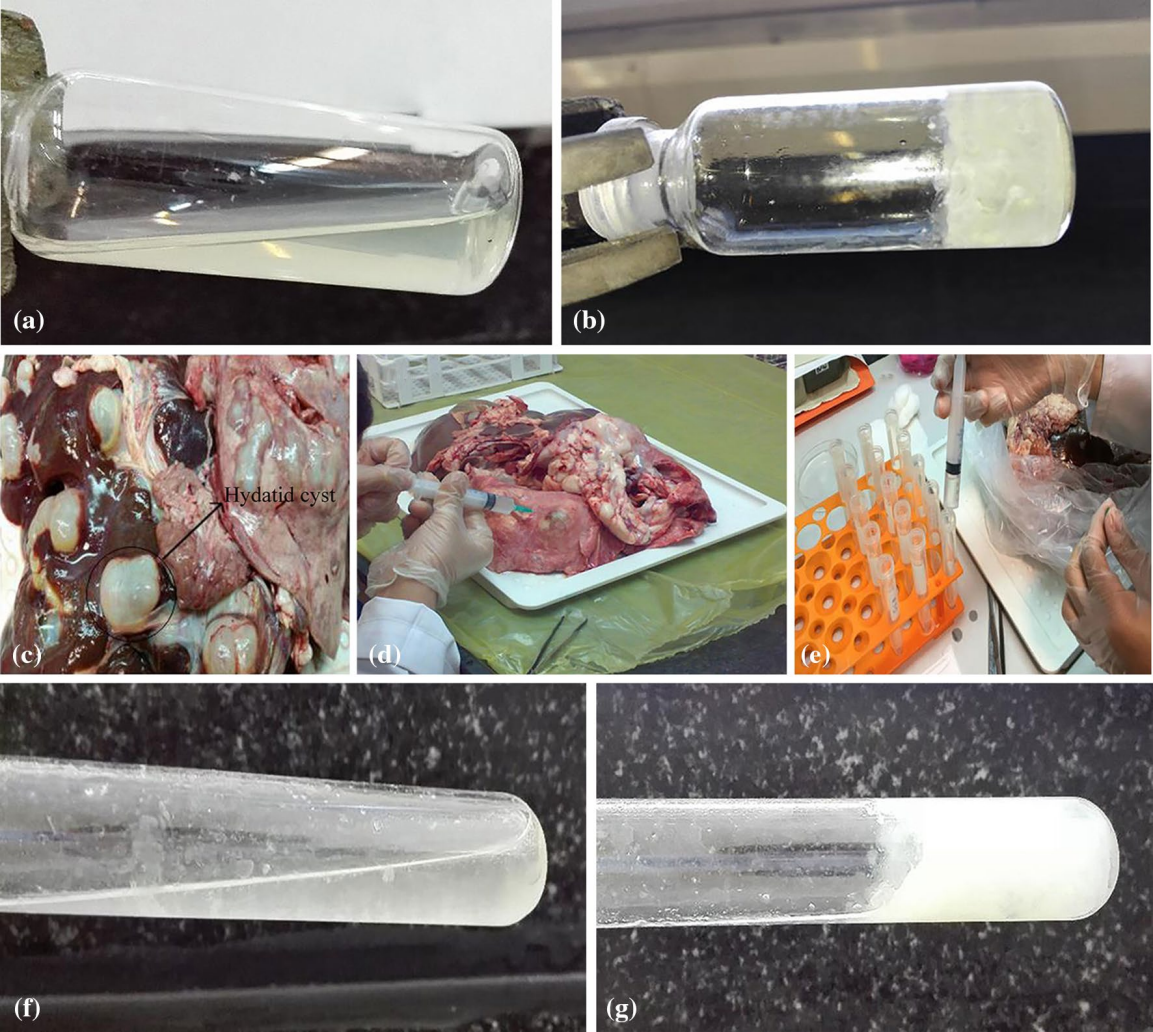
**a** Polymer solution at 4 °C with CS/CMC ratio of 1.75/1.4 and BGP (2.9%), **b** formed gel at 37 °C, **c** contaminated liver hydatid cyst, **d** aspirated HCF, **e** sterilized HCF. **f** Injected polymer solution with CS/CMC ratio of 1.75/1.4 and BGP (2.9%) to HCF (1:3) at 25 °C, **g** solidified HCF at 37 °C

The process of solidification of HCF is depicted in Fig. 10. Animal (sheep) hydatid cysts were collected from the liver of infected sheep from the local abattoir and brought to the laboratory of parasitology, School of Medicine at Shiraz University of Medical Sciences. Hydatid cyst fluid was aseptically aspirated and centrifuged at 1000*g* at 4 °C for 30 min. The supernatant was removed and stored at − 70 °C before use. For solidification, pre-frozen HCF was prepared and pre-chilled thermosensitive solution including CS/CMC/BGP with CS/CMC ratio of 1.75/1.4 was selected. The CS/CMC/BGP solution was injected with insulin needle to HCF with ratio of 1:3. The final BGP concentration was 2.9%. The main reason for the selection of this ratio for CS and CMC in the final blend hydrogel was based on gel strength and uniform microporous network in this case which was mentioned before. Finally its solidification took place for about 45 min at 37 °C (Fig. 10) which seems reasonable, but some modifications to help shorten the gelation time in HCF are needed. The longer gelation time in the presence of HCF might be linked to the physicochemical nature of the hydatid cyst fluid. Exploring and having the properties of HCF as well as salt ion type and salt concentration in HCF would indeed help to reduce the solidification time of HCF, because gelation behaviors of thermosensitive polymers are affected not only by the polymer concentration, but also the type of ions in aqueous solution (Wu et al. 2016).

### In vitro cytotoxicity

**Fig. 11:**
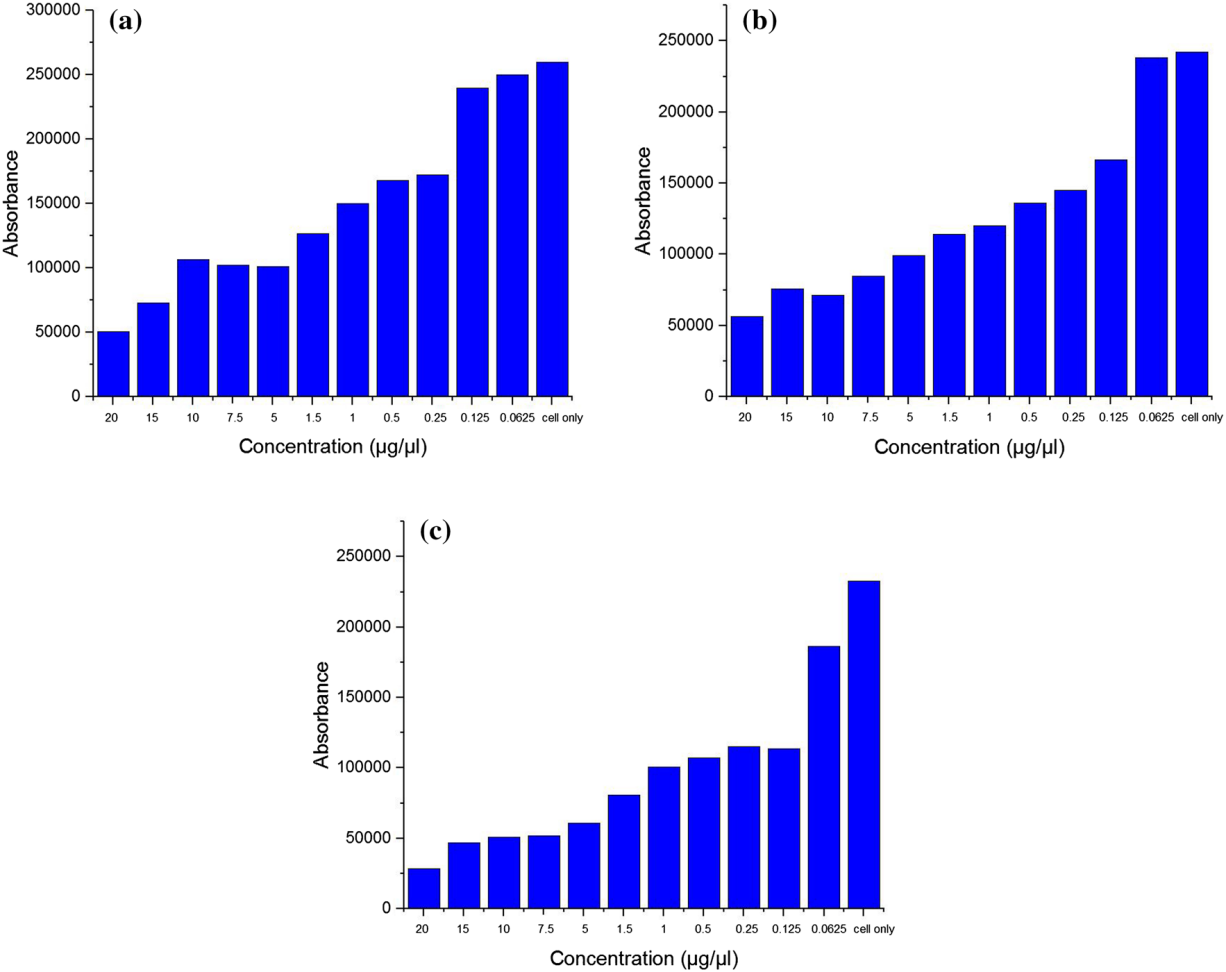
In vitro cytotoxicity results for CS (1.75%)/CMC (1.4%)/BGP (2.9%) polymer dilute solution. **a** Treatment for 24 h, **b** 48 h and **c** 72 h

The viabilities of the fibroblast cells grown were evaluated using the Alamar Blue assay according to the standard protocol (Al-Nasiry et al., 2007). First, a CS (1.75%)/CMC (1.4%)/BGP (2.9%) polymer solution was prepared as stock solution. Fibroblast cells were seeded at a density of 10^4^ cells per well in a 96-well tissue culture microplate. After 24 h of culturing, the cells were treated with diluted polymer solution (0.0625, 0.125, 0.25, 0.5, 1, 1.5, 5, 7.5, 10, 15 and 20 μg/μL) for 24, 48 and 72 h, respectively. Subsequently, 20 μL of Alamar Blue solution was added into each well. After incubating for 4 h at 37 °C, the plates were read with microplate fluorometer reader, using an excitation wavelength of 570 nm and an emission wavelength of 585 nm. The results are shown in Fig. 11, and the IC50 values were determined with GraphPad prism 5 software and represented in Table 5. IC50 values reveal that the polymer solution is non-toxic and safe for human cells (Taylor et al. 2014).

**Table 5:** IC50 values of CS (1.75%)/CMC (1.4%)/BGP (2.9%) for different times

Time (h)	IC50 values (μg/μL)
24	0.598
48	0.235
72	0.138

## Conclusions

Injectable and thermosensitive CS/CMC/BGP blends were prepared as physically cross-linked gel. From the FTIR analysis, hydrogen bonding between chitosan and CMC was established. SEM images depicted that freeze-dried hydrogels had a porous structure. Viscoelastic behavior showed that the prepared hydrogel had a phase transition of sol–gel near the body temperature. Water uptake results showed that appreciable swelling with a good strength was obtained. A polymer solution of CS/CMC/BGP with CS/CMC ratio of 1.75/1.4 was injected to HCF (1 mL polymer solution to 3 mL HCF) to reach a final concentration of 2.9% for BGP to solidify HCF. Results revealed that the solidification took place less than 45 min at 37 °C. Cytotoxicity results showed that the polymer solution was non-toxic. Therefore, the authors believe that CS/CMC/BGP blend has a great potential as an injectable hydrogel. Much work remains to be done, including shortening the solidification time of HCF, in vivo studies and evaluation of the inflammatory response. However, the door is open for studies of effective ways to control spillage during aspiration of hydatid cysts.
