# Correction to: Solidification of hydatid cyst fluid with an injectable chitosan/carboxymethylcellulose/β-glycerophosphate hydrogel for effective control of spillage during aspiration of hydatid cysts

**DOI:** 10.1007/s40204-018-0086-1

**Published:** 2018-04-07

**Authors:** Mostafa D. A. Azadi, Shadi Hassanajili, Khalil Zarrabi, Bahador Sarkari

**Affiliations:** 1 Department of Chemical Engineering, School of Chemical and Petroleum Engineering, Shiraz University, Shiraz, Iran; 2 Department of Cardiovascular Surgery, School of Medicine, Shiraz University of Medical Sciences, Shiraz, Iran; 3 Department of Parasitology and Mycology, School of Medicine, Shiraz University of Medical Sciences, Shiraz, Iran

## Correction to: Progress in Biomaterials (2018) 7: 35–54 10.1007/s40204-018-0082-5

The original version of this article unfortunately contained a mistake: the spelling of the Shadi Hassanajili’s name was incorrect. The corrected name is given above.

